# Supplementary material for: Is It Possible to Have Home E-Monitoring of Pulmonary Function in Our Patients with Duchenne Muscular Dystrophy in the COVID-19 Pandemic?—A One Center Pilot Study
Source: Int J Environ Res Public Health. 2021 Aug 26;18(17):8967. doi: 10.3390/ijerph18178967 (PMC8430665; doi:10.3390/ijerph18178967)
Supplement: Supplementary file 1 [file ijerph-18-08967-s001.zip › ijerph-1341493-supplementary.pdf]

Table S1. Survey of satisfaction and possibility of home e-monitoring in DMD patients.

**Home AioCare e-spirometry**				
**1.** Mark on the scale how you rate the monitoring of lung function with the home spirometer.				
1	2	3	4	5
the worst				the best
**2.** Mark on the scale how you assess the comprehensibility of the instructions received for self-measurements of spirometry.				
1	2	3	4	5
the worst				the best
**3.** If you have not been able to perform spirometric measurements regularly, what was the most common reason for this?				
- Correct blowing into the spirometer is too difficult for me				
- I don't have time to blow into the spirometer				
- I have no motivation for this assessment				
- I feel unwell				
- I forget to take the measurements				
- other reasons:				
**4.** What would be helpful for you to take measurements every day?				
- reminder sent via SMS				
- weekly online appointment with your doctor				
- weekly report of my results with interpretation				
- I am not able to take measurements everyday				

- other: ....
**5.** Do you think that you benefit from home spirometry measurements?
YES NO
**6.** If ‘YES’, the benefits are:
- I can breathe easier
- I can clear my lungs easier
- I feel more confident
- I like to use new electronic spirometer
- I will not be afraid of performing spirometry in the hospital
- other: ....
